# Prof. L. M. Lawson

**Published:** 1850-05

**Authors:** 


*Prof. L. M. Lawson.*—Prof. Lawson, editor of the Western Lancet, is about to sail for Europe, designing to pass the summer abroad, for professional purposes. Dr. G. Mendenhall, supplies his place, as editor of the Lancet, during his absence.

